# Smooth Muscle α Actin (*Acta2*) and Myofibroblast Function during Hepatic Wound Healing

**DOI:** 10.1371/journal.pone.0077166

**Published:** 2013-10-29

**Authors:** Don C. Rockey, Nate Weymouth, Zengdun Shi

**Affiliations:** 1 Department of Internal Medicine, Medical University of South Carolina, Charleston, South Carolina, United States of America; 2 Division of Digestive and Liver Diseases, University of Texas Southwestern Medical Center, Dallas, Texas, United States of America; University of Navarra School of Medicine and Center for Applied Medical Research (CIMA), Spain

## Abstract

Smooth muscle α actin (*Acta2*) expression is largely restricted to smooth muscle cells, pericytes and specialized fibroblasts, known as myofibroblasts. Liver injury, associated with cirrhosis, induces transformation of resident hepatic stellate cells into liver specific myofibroblasts, also known as activated cells. Here, we have used *in vitro* and *in vivo* wound healing models to explore the functional role of *Acta2* in this transformation. *Acta2* was abundant in activated cells isolated from injured livers but was undetectable in quiescent cells isolated from normal livers. Both cellular motility and contraction were dramatically increased in injured liver cells, paralleled by an increase in *Acta2* expression, when compared with quiescent cells. Inhibition of *Acta2* using several different techniques had no effect on cytoplasmic actin isoform expression, but led to reduced cellular motility and contraction. Additionally, *Acta2* knockdown was associated with a significant reduction in Erk1/2 phosphorylation compared to control cells. The data indicate that *Acta2* is important specifically in myofibroblast cell motility and contraction and raise the possibility that the *Acta2* cytoskeleton, beyond its structural importance in the cell, could be important in regulating signaling processes during wound healing *in vivo*.

## Introduction

Actin plays an important role in many cellular processes, including cell division, cell motility and the generation of contractile force. Eukaryotic cells contain at least six unique actin isoforms, encoded by a multigene family [1], [2]. Two nonmuscle or cytoplasmic actins, β and γ, are found in all cells while the muscle actins include γ smooth muscle actin, and 3 α actin variants (smooth, cardiac and skeletal), each of which is restricted to specialized muscle or muscle-like cells [3], [4]. The smooth muscle α actin (*Acta2*) isoform is found predominantly in smooth muscle, but is also expressed in other specialized cells such as pericytes and myofibroblasts, the latter of which are typical of wound healing [5]–[7].

From a structural standpoint, actins are among the most highly conserved proteins known (**Figure S1**). Despite the fact that the 6 known eukaryotic actin isoforms are coded for by 6 different genes, the actins exhibit remarkable amino acid similarity [8]. The group of muscle specific actins (smooth muscle γ and α actin, cardiac α actin, and skeletal α actin) differ from nonmuscle cytoplasmic actins at less than 10% of amino acid locations, while the muscle specific isoforms differ from each other only at several residues [1], [9], primarily at the amino-terminus [1], [2], [8], [9]. Considerable controversy exists regarding the degree that the minor variations in actin structure confer functional specificity among the isoactins [4], [10]. A weak interaction between actin and myosin which appears to be dependent on the negatively charged amino-terminal region of actin and the positively charged flexible loop on the myosin head [11] raises the possibility that differences in actin structure in the amino-terminal region could lead to divergent functional characteristics of the actins.

Persistent injury leads to a wounding response, common to many tissues and typified by fibrogenesis as well as wound contraction [6], [12]–[16]. A key feature of the cellular response to injury, regardless of tissue type, is the appearance of a population of specialized cells known as myofibroblasts [17], [18]. In the liver, injury and the subsequent wounding response leads to activation of resident mesenchymal cells known as hepatic stellate cells [19]–[21] which undergo a programmed cascade of events, including enhanced matrix synthesis, cellular proliferation, and striking de novo production of *Acta2* [13], [21], [22]. The stellate cell to myofibroblast transformation process, also known as “activation” - in which *Acta2* is an integral component - appears to be analogous to that occurring in fibroblasts after injury and wound healing in other pathological settings [7], [23]–[27].

In this study, we hypothesized that *Acta2*, which is upregulated during stellate cell activation, has a critical functional role in stellate cell phenotypic behavior during the wound healing response. In particular, cell motility and contractility appear to be stellate cell phenotypes important during the wounding response. Thus, we have utilized *in vivo* models of liver injury with primary stellate cells, including those isolated directly from injured livers. This activation response resulting from injury causes stellate cells to transform into myofibroblast-like cells and allows us to more accurately explore the functional role of *Acta2* in cell motility and contractility. This model in particular yields a more accurate assessment of *in vivo* cellular behavior than systems utilizing passaged or transformed cells.

## Results

### Actin isoform regulation in hepatic stellate cells during hepatic wounding

**Figure 1:**
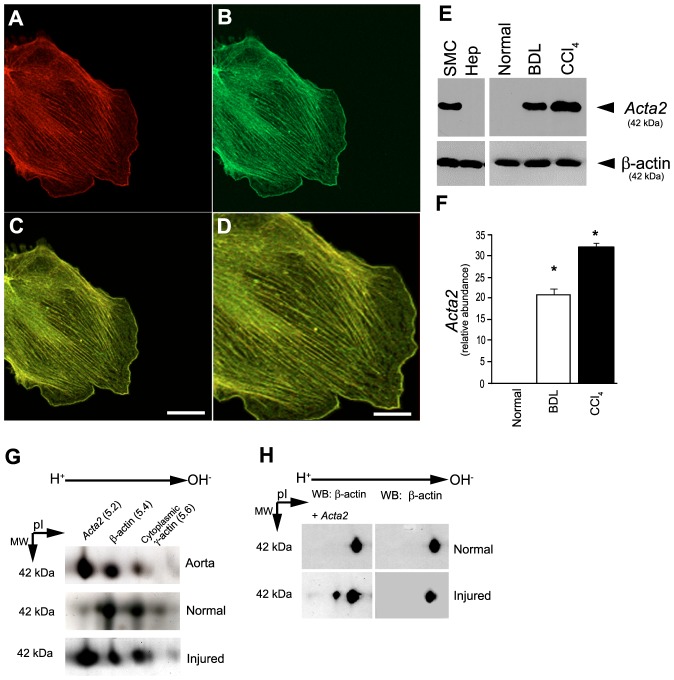
Actin isoform expression after liver injury. In (A–C), stellate cells were isolated after carbon tetrachloride (CCl_4_) induced liver injury as in Methods and plated on glass coverslips. Twenty-four hours later, smooth muscle α actin (*Acta2*) (A, Texas red) and nonmuscle β-actin (B, FITC) were detected by immunocytochemistry as in Methods. In (C and D) are shown overlays, revealing co-localization of actins (C: bar = 10 microns; D: bar = 5 microns). Identical results were obtained with cells after either form of liver injury, and images are representative of over 20 others. In (E), stellate cells were isolated from normal livers or 8 days after bile duct ligation or 10 doses of carbon tetrachloride and immediately subjected to immunoblotting as in Methods. Representative immunoblots shown depict duplicate, identical, samples probed for each *Acta2* and anti-cytoplasmic β actin (7.5 µg total protein). In (F), specific bands were scanned, quantitated and expressed graphically (n = 4 for each model of injury, *p<0.001 compared to normal). In (G), stellate cells from normal or injured livers were immediately lysed and equal amounts (40 µg) of cellular proteins were subjected to 2-D gel electrophoresis as in Methods. Notably, we also made a theoretical estimation of isoactin PIs by in silico analysis of each actin isoform ([67](**Figure S1**)). Representative examples (of greater than 20 separate experiments) reveal specific actin isoforms, and after injury (bile duct ligation), new expression of an α isoform (two-D gels are shown in the standard international format with pI ranging from acidic to basic, left to right). In (H), a representative immunoblot of similarly prepared protein samples after 2-D gel electrophoresis is shown (200 µg total protein each). As described in Methods, nitrocellulose membranes were probed sequentially with anti-cytoplasmic β-actin then anti-*Acta2* (using the same ECL detection method each time, thus accounting for repeat detection of the β-actin band). Abbreviations: *Acta2* - smooth muscle α actin; BDL - bile duct ligation; CCl_4_ - carbon tetrachloride.

Our model system exploits our ability to isolate in high purity and to examine primary rat stellate cells after induction of liver injury; by all accounts, their study immediately after their isolation provides a very close approximation of their *in vivo* phenotype [22]. We first evaluated actin, including *Acta2* expression in two models of hepatic injury and wounding (**Figure 1**). Repeated administration of carbon tetrachloride (10 doses over 70 days) and bile duct ligation led to prominent stellate cell activation, expression of *Acta2*, and fibrosis as described [22].

Given previous reports of the dramatic upregulation of *Acta2* after liver injury [22], we examined regulation of this and other actin isoforms in this process. In individual stellate cells isolated immediately after liver injury, actin isoforms localized predominantly to stress fibers (**Figure 1A–D**), although small amounts of both *Acta2* and cytoplasmic β-actin isoforms were found at leading edges of migrating cells (**Figure 1C, D**). We further investigated isoactins in stellate cells by immunoblotting and 2-dimensional gel electrophoresis (**Figure 1E–H** **, Figure S1**). Levels of cytoplasmic β-actin did not appear to change after activation while levels of *Acta2* increased (**Figure 1E, F**). By 2-D gel electrophoresis, signals for cytoplasmic β and γ actin remained essentially unchanged after liver injury, while the signal corresponding to α actin appeared *de novo* after activation (**Figure 1E–H**). Immunoblotting of isoactins after 2-D gel electrophoresis with actin isoform specific antibodies verified that the signal corresponding to β actin was nonmuscle cytoplasmic β-actin and that corresponding to α actin was *Acta2* (**Figure 1H**). In aggregate, the data demonstrate that injury and wounding did not induce changes in cytoplasmic isoactins, but led to a significant increase in *Acta2* expression.

### Myofibroblast motility and contraction are enhanced during hepatic wounding

**Figure 2:**
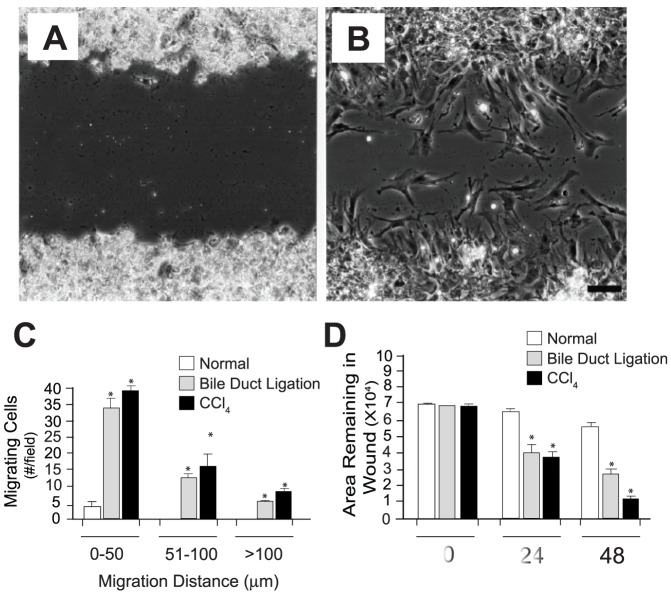
Enhanced stellate cell motility after liver wounding. Stellate cells isolated from normal and injured livers were isolated, plated at equal density and allowed to adhere in culture overnight. A linear scratch was applied to the monolayer and cell motility was assessed by phase contrast microscopy (A–B) and by quantitative analysis of cell movement into the scratch-wounded area (C–D). Photomicrographs shown in (A) and (B) depict examples of cells from normal liver (A) and after carbon tetrachloride injury (B) as in Methods; photomicrographs were taken after 24 hours and are representative of 15 different experiments (bar = 60 microns). In (C), cells entering the wounded area of the monolayer over 24 hours were counted (i.e., the number of cells moving the specified distances into the wounded area per high powered field were quantitated as in Methods, n = 6 for each model of injury). In (D), the area in the scratch remaining unoccupied by cells was quantitated (in each experiment, 10 random fields were assessed; the area remaining free of cells was measured by image analysis as in Methods, single data points were created for each experiment and were used to generate quantitative data; n = 6 for each model of injury). For (C) and (D), *p<0.001 compared to normal. Abbreviations: CCl_4_ - carbon tetrachloride.

Stellate cells were isolated and subjected to linear scratch wounding assays as in Materials and Methods. Cells isolated from normal animals remained relatively compact and had typical prominent retinoid inclusions (**Figure 2A**); note that the abundant retinoid droplets remain in a highly compact fashion after early isolation, and cause the cells to take on a refractile appearance when viewed by phase contrast microscopy. Cells from normal livers rarely entered the scratched area - even 48 hours later (**Figure 2A**). In contrast, cells from injured livers appeared activated, and myofibroblastic - containing less retinoid, and being markedly spread, were highly motile (**Figure 2B–D**). Not only did activated cells move into the scratch in a more rapidly than those from normal livers, but migration of cells >50 µm was identified only in cells isolated from injured livers (**Figure 2C**); quantitation of cell movement by image analysis further established the enhanced motility of cells from injured livers compared to normal cells (**Figure 2D**). Time-lapse video microscopy demonstrated that stellate cells from injured livers at the leading edge of the scratched area migrated at a rate of 4–7 µm per hour, while those from normal livers were essentially immobile over the initial 24 hours.

**Figure 3:**
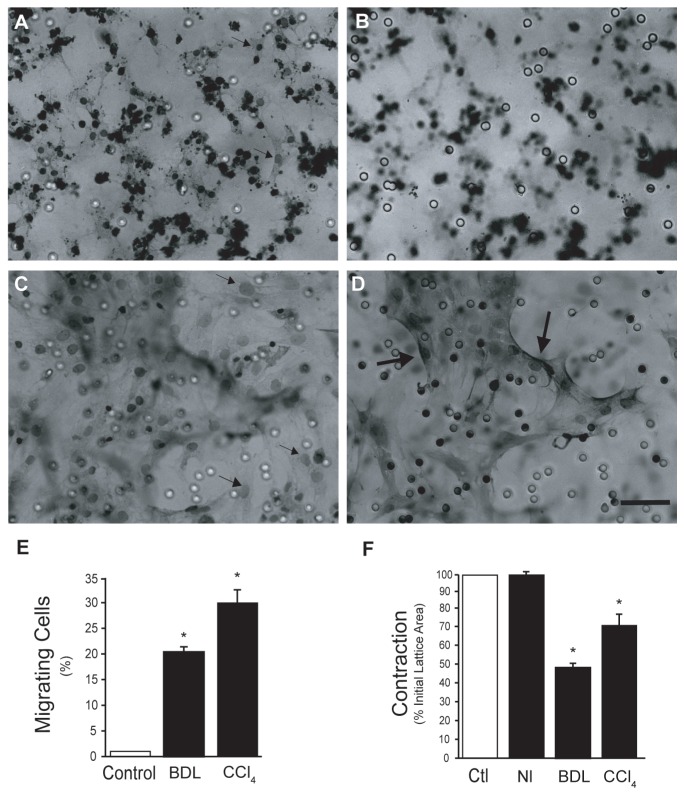
Enhanced migration and contraction of stellate cells after liver injury. Cells from normal and injured livers were isolated as in Methods and allowed to adhere on top of polyethylene terphthalate membranes containing 8 µm pores. Cells were plated in serum free medium; serum containing medium was placed in the bottom of transwell chambers. After 12 hours, membranes were washed, fixed with 4% paraformaldehyde and stained for 30 minutes with 0.4% hematoxylin. In (A) and (B) are shown representative examples of cells from normal liver and in (C) and (D) are shown cells from injured liver (carbon tetrachloride). Panel (A) shows an exposure focused on the top of the membrane, (B) depicts the same field, but focused on the bottom of the membrane. In (A), many cells remain compact and therefore are darkly stained, the small arrows point to cells that have begun to spread on the top of the membrane. In (B), no cells have passed through the membrane and therefore none are in focus. In (C) and (D) virtually all cells have spread markedly, the small arrows in (C) point to cells that have spread on the top of the membrane. In (D), the larger arrows point to cells that have migrated through the membrane (bar = 50 microns). In (E), the number of cells migrating to the bottom of the membrane were quantitated and expressed as a proportion of all cells plated (n = 4 for each model of injury, *p<0.001 vs. control (normal cells)). In (F), stellate cells from normal and injured livers were isolated and allowed to adhere on top of collagen lattices. After adherence for 18 hours, serum free conditions were introduced and medium containing endothelin-1 (2 nM) was added. Lattices were dislodged and contraction after 4 hours is shown (n = 4 for each injury model, *p<0.001 vs. control (normal cells)). Abbreviations: BDL - bile duct ligation; CCl_4_ - carbon tetrachloride; Nl - normal; Ctr – control.

To further test cell motility, migration of stellate cells was assessed using track etched polyethylene terphthalate membranes containing 8 µm pores. Again, cells isolated from normal livers largely remained compact, evidenced by the darkly stained nuclei and sparse cytoplasm (**Figure 3A**); these cells exhibited almost no trans-membrane motility over 12 hours (**Figure 3A, B, E**), while cells from injured livers spread rapidly and readily migrated across membranes (**Figure 3C–E**). Twenty-four hours after isolation, 30.3% and 20.1% of cells from livers wounded with carbon tetrachloride and by bile duct ligation, respectively, migrated through membrane pores, while we could identify almost no cells isolated from normal livers migrating through membrane pores.

We next examined cellular contractility after hepatic wounding. Again, cells early after isolation were studied, prior to culture-induced changes or potential artifact, so as to allow a direct analysis of their *in vivo* phenotype. Stellate cells from normal livers did not contract in response to serum (not shown) or endothelin-1 while those after injury and activation were highly contractile (**Figure 3F**).

### Correlation of actin isoform regulation with cell motility in hepatic stellate cells during hepatic wounding

**Figure 4:**
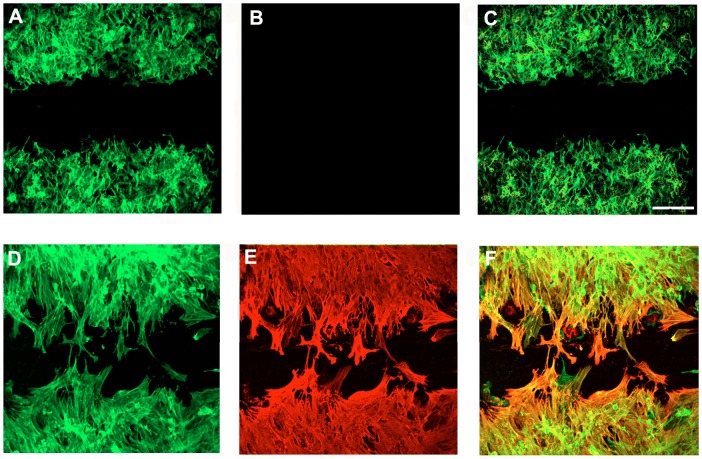
*Acta2* expression in normal and injured stellate cells during cell migration. Stellate cells from normal and injured liver (carbon tetrachloride) were isolated, plated at equivalent density and allowed to adhere in culture overnight as in Figure 1. After 12 hours, a linear scratch was applied to the monolayer. Twenty-four hours later, cells were fixed and dual labeled with anti-cytoplasmic β-actin and anti-*Acta2* antibodies as in Methods. In (A, cytoplasmic β-actin) and (B, *Acta2*), representative examples of cells from normal livers after scratch wounding are shown. In (D, cytoplasmic β-actin) and (E, *Acta2*), cells from carbon tetrachloride treated animals are shown. In C and F, co-localization of β-actin and *Acta2* is depicted in overlays. Representative areas from typical experiments (carbon tetrachloride) are shown (n = 15) (bar = 100 microns).

In a scratch wounding assay, stellate cells from normal livers were relatively immobile (**Figure 4A–C**), consistent with data in **Figures 2** **and** **3**, and moreover expressed only cytoplasmic (β) actin (the staining pattern for F-actin was identical to cytoplasmic β-actin). In contrast, cells from injured livers were highly motile, and expressed both *Acta2* and cytoplasmic β-actin (**Figure 4D–F**) (again, staining for F-actin was identical to that for cytoplasmic β actin). Of note, cells migrating into the scratched areas appeared to exhibit more intense *Acta2* labeling than cytoplasmic β-actin expression (**Figure 4F**); this was verified by demonstrating that quantitative fluorescence intensity in cells migrating into scratch-wounded areas was greater for *Acta2* than for cytoplasmic β-actin.

### Inhibition of Acta2 expression impairs cell motility and contractility

The parallel upregulation of *Acta2* and increase in stellate cell motility and contractility during activation suggested a specific functional role for *Acta2* in these processes. Thus, to specifically address the role of *Acta2* in motility and contractility, we used 2 different approaches. First, we utilized a well characterized primary cell culture model system in which stellate cells isolated from normal livers are placed on plastic or glass substratum and in the presence of serum, subsequently undergo spontaneous activation, transforming into myofibroblasts. Secondly, we examined cell motility of mouse embryo fibroblasts and stellate cells that did not express *Acta2*.

**Figure 5:**
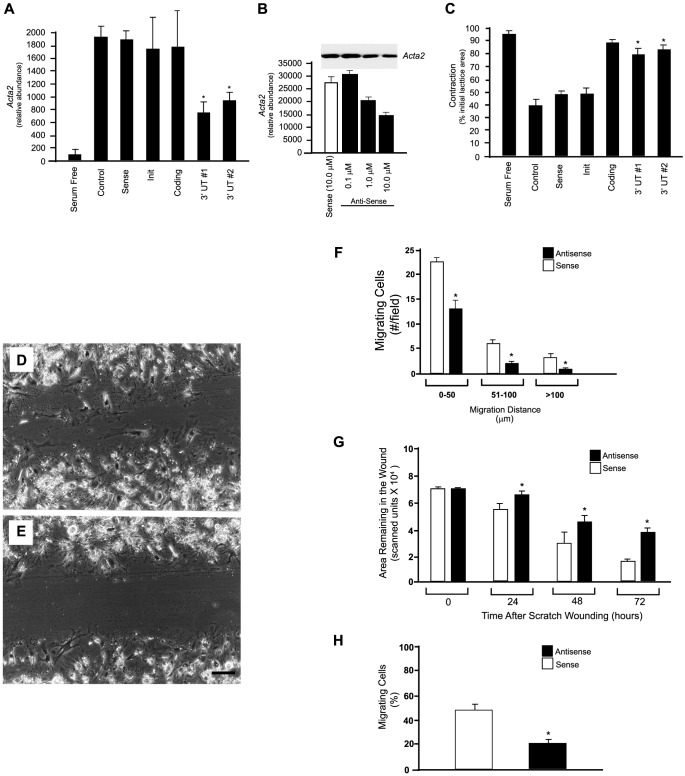
*Acta2* antisense oligodeoxynucleotides inhibit *Acta2* expression, stellate cell contractility, and stellate cell motility. Stellate cells were isolated from normal rat livers; after 24 hours, oligonucleotides were transfected as in Methods (the transfection mix containing oligonucleotides was replaced every 48 hours). Five days later, cells were harvested and lysates were subjected to immunoblotting to detect *Acta2*. In (A), different oligonucleotides (10 µM) were tested; specific *Acta2* bands were scanned, quantitated and expressed graphically (n = 3, * p<0.01). In (B), the effect of different concentrations of sense and antisense oligonucleotides (the *Acta2* 3′UT #1 sequence) was examined. The upper portion of the figure depicts a representative immunoblot, and the graph below depicts scanned and quantitated data (n = 3, * p<0.01). Immunoblots with anti-cytoplasmic β-actin revealed no change in *Acta2* expression (not shown). In (C), cells as above were placed on collagen lattices; oligonucleotides were added 24 hours later (all at 10 µM) and replaced at day 3 and 5 in culture. Serum free conditions were introduced and medium containing serum (10% horse/10% calf) was added to induce contraction. Lattices were dislodged from their plastic substrata and gel contraction was measured (contraction after 4 hours is shown, n = 4, *p<0.01 compared to lattices exposed to sense oligonucleotides). Cells exposed to only serum free or serum containing medium served as negative and positive controls, respectively. In (D–H), stellate cells from normal livers were isolated and allowed to undergo culture induced activation. Twenty-four hours after isolation, cells were transfected with oligodeoxynucleotides as in Methods. Seventy-two hours later, a linear scratch was applied to the cell monolayer. In (D), cells exposed to 3′UT #1 sense oligonucleotides (10 µM) are shown; in (E) cells exposed to 3′UT #1 antisense oligonucleotides (10 µM) are shown (representative images 24 hours after scratch wounding are shown) (bar = 50 microns). In (F), the number of cells per high-powered field entering the wounded area of the monolayer were counted and quantitated as in Methods (n = 6, *p<0.01 vs. cells exposed to sense oligonucleotides). In (G), the area in the wound remaining unoccupied by cells was quantitated by image analysis as in Methods (n = 6, *p<0.01 vs. cells exposed to sense oligonucleotides). In (H), the effect of *Acta2* antisense oligodeoxynucleotides on stellate cell motility was assessed by measuring migration of stellate cells through polyethylene terphthalate membranes containing 8 µm pores as in Figure 2 (n = 3, *p<0.01 vs. to sense). Abbreviations: Init - initiation; UT – untranslated.

In the stellate cell culture-based model system, which mimics activation *in vivo*, *Acta2* is absent in cells isolated from normal liver as in **Figure 4**; *Acta2* mRNA expression becomes upregulated during early culture and *Acta2* filaments are detectable within 72 hours after initial plating; the level of *Acta2* expression continues to increase over time in primary culture in the presence of serum or appropriate agonist [22]. In this model system, we continuously exposed stellate cells to *Acta2* antisense oligodeoxynucleotides (oligos). Multiple antisense oligos coding for sequences in different portions of the *Acta2* gene were examined, but we focused on the 3′ untranslated (UT) region for 2 reasons. First, this portion of the gene is the least well conserved among the actins [3] and targeting it would in theory be most specific. Secondly, previous reports have pointed to this region as selective for the actins [28], [29]. Sequences in the 3′ UT region had the most potent inhibitory effect (**Figure 5A**); other sequences tested did not have significant inhibitory effects. Further, *Acta2* 3′UT #1 antisense oligos exhibited a dose-response effect on *Acta2* expression (**Figure 5B**). Because the actin family is highly conserved, we examined whether 3′UT #1 antisense oligos had effects on cytoplasmic β-actin in stellate cells; immunoblot analysis revealed no effects of this antisense oligo on cytoplasmic β-actin. Further, immunocytochemical studies demonstrated that *Acta2* sense oligos had no effect on *Acta2* or cytoplasmic β-actin. Additionally, we found no effect of the *Acta2* antisense oligos on cytoplasmic β or γ actin mRNA or protein expression.

We next examined the effect of 3′UT #1 antisense oligos on stellate cell contractility and motility. Antisense oligos directed at the 3′ UT areas significantly reduced stellate cell contraction, while controls had no effect (**Figure 5C**). In the *in vitro* scratch wounding assay system, 3′UT #1 sense oligodeoxynucleotides had no effect on cell motility compared to controls in which no oligodeoxynucleotides were added while antisense oligodeoxynucleotides significantly reduced stellate cell motility (**Figure 5D–G**). Inhibition of *Acta2* also reduced the proportion of cells migrating through polyethylene terphthalate membranes by 43% compared to sense oligos, while migration of cells exposed to sense oligos (**Figure 5H**), and all appropriate controls was not affected. Importantly, all cells migrating through the polyethylene terphthalate membrane expressed *Acta2*, whether exposed to sense or antisense oligos (n = 4 for each), further supporting a link between *Acta2* and cell motility.

Immunocytochemical studies further revealed that *Acta2* 3′UT #1 antisense oligos inhibited both *Acta2* expression and motility while sense oligos had no effect (**Figure S2A–F**). Interestingly, cells migrating into the scratch wound exhibited the highest relative levels of *Acta2* expression (**Figure S2F**). To help quantitate the relative abundance of each specific isoform after exposure to oligos, we measured β-actin and *Acta2* fluorescence intensity. Although β-actin intensity did not change after exposure to antisense oligodeoxynucleotides, that for *Acta2* decreased several-fold.

**Figure 6:**
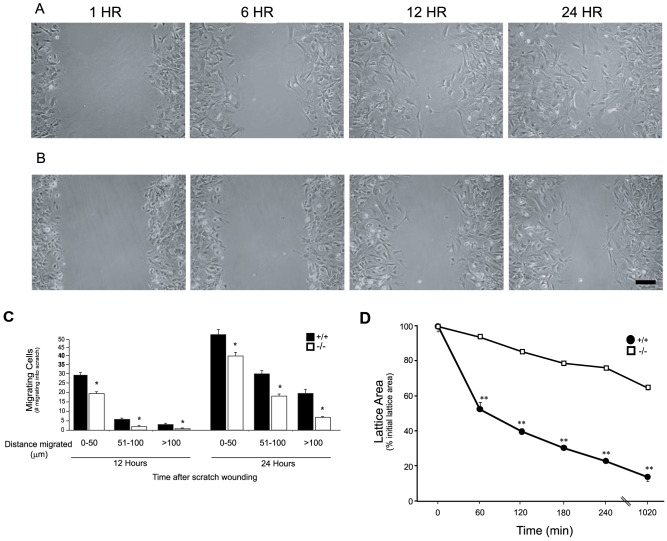
Reduced cellular motility and contractility in *Acta2* deficient cells. *Acta2* wild type (+/+) and null (−/−) fibroblasts were isolated from mouse embryos as in Methods. At the second to sixth passage, cells were plated in monolayers at uniform density and subjected to scratch wounding as in Methods. In (A) (+/+) and (B) (−/−), representative examples of cells migrating into scratched areas at different times are shown. In (C), cells migrating the specified distances and 12 and 24 hours after scratch wounding were counted (n = 6, *p<0.01 for +/+ vs. −/− cells). In (D), stellate cells from *Acta2* deficient (−/−) and wild type (+/+) were placed on top of collagen lattices and contraction was measured as in Methods (n = 4, **p<0.005 for +/+ vs. −/− cells).

To further explore the role of *Acta2* in cell motility, we also examined cells from *Acta2* deficient mice [30]. Actin isoform expression in these cells was studied extensively. We did not identify significant changes in the heterologous actins – cytoplasmic β-actin, cytoplasmic γ-actin, smooth muscle γ and α actin, cardiac α actin, or skeletal α actin - in *Acta2* deficient cells at the mRNA or protein level compared to wild type cells. We evaluated cell motility in *Acta2* deficient mouse embryo fibroblasts (MEFs) and in stellate cells isolated from these mice. Functional assays of *Acta2* deficient MEFs revealed that they exhibited reduced motility compared to wild type cells (**Figure 6A–C**); we also performed studies of mouse stellate cell motility and found that their motility phenotype was identical to MEFs; thus, due to the technical difficultly in obtaining large numbers of stellate cells and since the profiles of activated stellate cells and MEFs were identical, we performed multiple replicate functional studies in the latter only. Additionally, MEFs lacking *Acta2* also exhibited a reduced contraction phenotype (**Figure 6D**). Of note, *Acta2* +/+ MEFs grown in the presence of 10% FBS expressed *Acta2* in stress fibers, while as expected, −/− MEFs did not, and both cell types expressed cytoplasmic β-actin, again in stress fibers.

### *Acta2* activates Erk

**Figure 7:**
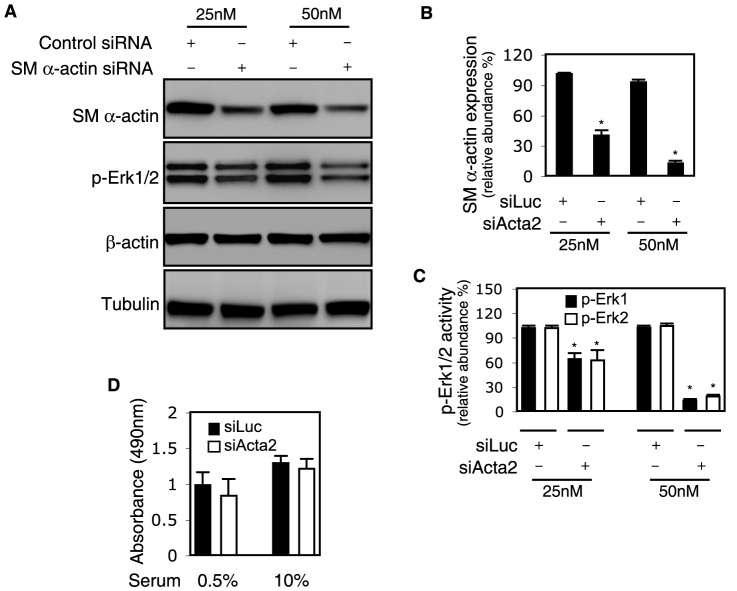
*Acta2* and Erk signaling. In (A), rat stellate cells were isolated and grown in standard medium for 2 days as described in methods and then exposed to smooth muscle (SM) α actin (*Acta2*) siRNA (siActa2) or control siRNA (siLuc) for 48 hours as in Methods. Cells were incubated in 0.5% serum medium for a further 24 hours and then harvested. Equal quantities of protein lysate (25 µg) were subjected to immunoblotting to detect the identified proteins and representative images are shown; quantitative data are presented graphically (B and C, n = 3; *p<0.05 for siLuc vs. siActa2). In (D), stellate cells as above were seeded at a density of 1×10^4^ per well in 96 well plates and transduced siRNA siActa2 or control siRNA siLuc for 48 hours and then incubated in 0.5% or 10% serum medium for a further 24 hours. Cell proliferation was measured as described in Methods, with proliferation being proportional to absorbance. Abbreviations: SM - smooth muscle; siActa2 - smooth muscle α actin or *Acta2* siRNA; siLuc - luciferase siRNA.

The Erk MAPK pathway plays a critical role in a variety of cellular processes, including migration, contraction, and proliferation [31], [32]. Thus, we asked whether the *Acta2* cytoskeleton could be important in regulation of Erk signaling. First, we demonstrated that siRNA mediated knockdown of *Acta2* was feasible (**Figure 7A** **, top panel**). Additionally, there were no significant changes in other actin isoform mRNA expression (i.e. the cytoplasmic actins, smooth muscle γ and α actin, cardiac α actin, or skeletal α actin –**Figure S1**) in *Acta2* knockdown cells compared to controls.

Knockdown of *Acta2* (**Figure 7A** **, top panel and** **Figure 7B**) paralleled a significant reduction in Erk1/2 phosphorylation (**Figure 7A** **, second panel and** **Figure 7C**); there was no effect on β-actin or tubulin. These data suggested that *Acta2* regulates Erk activity during stellate cell activation. Interestingly, while Erk activity during stellate cell activation has been reported to important in stellate cell proliferation [33], *Acta2* knockdown did not affect stellate cell proliferation, when stimulated with a high concentration of serum (**Figure 7D**).

## Discussion

We show here that *in vivo* stellate cell activation after liver wounding is associated with a striking increase in cellular motility and contractility; this functional transition parallels an increase in expression of *Acta2*, typical of myofibroblasts. Additionally, inhibition of *Acta2* expression (with many different methods) reduced both stellate cell motility and contractility.

Our data raise important issues regarding actin isoform structure and function. On one hand, we have shown that *Acta2* is important in cellular contractility as well as motility, functions that have often been attributed to nonmuscle isoforms. Despite the normal expression of non-muscle actins, we have shown that a lack of *Acta2* significantly impairs cell motility (**Figures 2** **–** **4** **,** **6**), raising the possibility of functional specificity. Further, contraction in *Acta2* null cells is compromised, consistent with previous observations [34]–[41]. On the other hand, we cannot rule out the possibility that *Acta2* supports motility and contractility by contributing to the total actin pool. Additionally, the finding that *Acta2* null cells retained some measure of contractility and motility suggests functional redundancy for actin, which is not surprising given the remarkable sequence conservation among the actin isoforms [4], [10]. An abundance of cell-based and whole organism-based literature support the existence of each isoactin functional specificity and redundancy [34]–[41]. Therefore, based on these previous data, and our own work, we conclude that a complex interplay of isoactin expression and dynamics at the cellular level is likely to determine the functional fate of each actin.

Previous reports examining *Acta2* and general cellular contractility are in agreement with our findings while one studying cellular motility is not. It was shown that inhibition of *Acta2* expression reduced cell force generation [42] and gingival fibroblast mediated collagen gel contraction [43], consistent with our findings and also supporting the position that *Acta2* functions as a contractile protein. In another report, it was suggested that *Acta2* functions as a “brake” for motility [28]. In this study, fibroblasts derived from clonal expansion of cell lines expressing *Acta2* were less motile than lines lacking *Acta2*. However, we found upregulation of *Acta2* to be associated with enhanced motility and that deletion of *Acta2* null fibroblasts led to reduced motility compared to wild type cells expressing increased amounts of *Acta2*. Although the previous study and our own would appear to be paradoxical, several points merit emphasis. First, our study characterized *Acta2* in cells isolated directly from a normal or injured organ; their behavior is more likely to mimic that occurring *in vivo*. In contrast, in the previous study, cloned and highly selected fibroblast cell lines were examined. Although changes in *Acta2* expression were well characterized, it is unknown whether changes in expression of other proteins that could affect cell motility were introduced during clonal expansion.

Our data are consistent with other data in stellate cells that have emphasized a prominent motility phenotype specifically in this cell type. In one study, migration of stellate cells increased after injury, but deletion of moesin significantly reduced cell motility [44]. In another study, it was likewise shown that activated stellate cells were motile [45], and additionally that inhibition of the myosin II ATPase with blebbistatin, stimulated stellate cell migration. Finally, it was demonstrated that a microtubule-destabilizing protein found in neurons, SCG10, was upregulated in stellate cells after injury [46], highlighting a potential mechanism for enhanced stellate cell migration after liver injury.

Understanding the function of specific cytoskeletal proteins is inherently difficult because collective cytoskeletal behavior depends on the complex arrangement and interaction of many components, all of which ultimately play a role. This is particularly relevant in our system since stellate cells undergo activation after injury, and the activation process almost certainly modifies multiple elements of the cytoskeleton. Thus, while we believe that *Acta2* is important in stellate cell contraction and motility, other factors are also likely to be critical. For example, we have found that α-actinin, an actin linking protein, is highly expressed in stellate cells during activation; further, it has been shown that myosin heavy chains, which serve as motors for motility, are also present in activated stellate cells [47]. In addition, cell motility and contractility are linked with multiple molecular pathways [46], [48]–[51]. We have previously demonstrated increases in Rho associated kinase (ROCK) and ROCK activity [52] and other signaling cascades after activation [52], [53], which are involved in organizing the actin cytoskeleton needed for cell contraction and motility. Here, we have further demonstrated that *Acta2*, and presumably the actin cytoskeleton, is important in regulation of Erk (**Figure 7**). It is commonly accepted that Erk plays a critical role in cell motility and contraction through phosphorylation of FAK, calpain-2, paxillin, MLCK, and other signaling partners [32], [54]. Thus, our data suggest that reduced motility and contractility in *Acta2* deficient stellate cells appears at least in part to be due to reduced Erk activity. Interestingly, *Acta2* did not appear to be a prominent regulator of stellate cell proliferation (**Figure 7**). We speculate that these complex systems, including interaction of signaling partners, extracellular matrix binding proteins (i.e. integrins), turnover of focal adhesions, as well as the actin cytoskeleton are all likely to be important in mediating stellate cell migration and motility during wound healing.

In summary, wound healing is a dynamic process in which cell migration and contraction are important components [55], [56]. Myofibroblasts, which share the unique property that they express *Acta2* during the wounding response, appear to be central to the process [23], [24], [57]–[60]. Further, our findings suggest that *Acta2* is critical for both cell motility and contractility, and thus plays an important role in myofibroblast function.

## Materials and Methods

### Ethics Statement

All animals received care according to NIH guidelines and the University of Texas Southwestern and the Medical University of South Carolina Institutional Animal Care and Use Committees (IACUC) approved the protocols.

### Liver Injury

Hepatic wounding was induced in male Sprague-Dawley rats (450–550 gram) by repetitive intragastric administration of carbon tetrachloride (10 weekly doses) or by bile duct ligation (for 14 days) as described [61]–[63]. Controls received corn oil or underwent sham laparotomy on the same schedule as experimental animals.

### Cell isolation and culture

Stellate cells were isolated from normal and injured male Sprague-Dawley rat livers (450–550 grams) as well as *Acta2* deficient (a kind gift from Dr. Robert Schwartz [30]) and wild type littermate mice as described [63], [64]. Stellate cells were greater than 99% pure as assessed by desmin immunoreactivity and intrinsic vitamin A autofluorescence.

### Motility and migration assays

Cells from normal or injured livers were isolated and cultured in confluent monolayers. After culture for a designated time period, a scratch was applied to the monolayer with a sterilized circular metal tip and cultures were maintained at 37°C. Cell migration was measured in a blinded fashion by (1) counting individual cells migrating specific distances into the linear scratched area using a calibrated grid reticle in the eyepiece (10 random fields were examined for each condition) and (2) by image analysis (in 10 random fields, the area remaining unoccupied by cells was measured) using NIH image. Photomicrographs were with a Nikon TE 300 photomicroscope (Nikon Co.), Nikon N6006 automatic camera (Nikon Co.) and Tmax film (Eastman Kodak Co., Rochester, NY).

To measure cell migration through membranes, cells from normal or injured livers were isolated and cultured in track etched polyethylene terphthalate membranes cell culture inserts with 8.0 µm pores. After the specified time period, inserts (both sides) were washed, fixed (4% paraformaldehyde), stained with 0.4% hematoxylin (Sigma), and mounted. For some experiments, inserts were fixed and processed for immunocytochemical studies as above.

### Immunocytochemistry

Cell cultures were washed with PBS and fixed with fresh paraformaldehyde (4%) in PBS, then 0.3% Triton X 100. After washing, cells were incubated overnight at 4°C in PBS containing anti-*Acta2* antibody (Clone 1A4, Sigma) diluted 1∶200, and Oregon Green conjugated phalloidin (Molecular Probes). Cells were washed and incubated with biotinylated anti-mouse IgG (Amersham) for 2 hours. In some cultures, cells were co-labeled with FITC conjugated anti-cytoplasmic β-actin antibody (Sigma), rather than with Oregon Green conjugated phalloidin. After washing with PBS, samples were incubated with streptavidin-linked Texas Red (Amersham) for 30 minutes, washed again and mounted. Photomicrographs taken with a Nikon TE 300 photomicroscope (Nikon Co.), Nikon N6006 automatic camera (Nikon Co.) and Ilford Plus film (Ilford Co.). In some experiments, confocal images were obtained with an 410 LSM Zeiss microscope (Carl Zeiss, Inc.); fluorescence intensity (I) measurements were obtained from entire cells and analyzed with Zeiss LSM 410 software. Control specimens were identical to experimental specimens except they were exposed to irrelevant isotype matched antibody.

### Two-dimensional gel electrophoresis

Cells were washed and lysed in buffer containing 0.3% SDS, 200 mM DTT, 28 mM Tris HCl and 22 mM Tris base at 100°C; nucleic acids were removed with RNase and DNase (Gibco BRL) and protein precipitated with 80% v/v ice cold acetone for 20 minutes. Samples were centrifuged and the pellet resuspended in sample buffer and equal amounts of protein were loaded onto pre-cast pH 4–8 carrier ampholyte tube gels (Genomic Solutions) and focused for 17 hours at 2,000 volts. SDS-PAGE of tube gels was carried out in precast 22×22 cm 10% acrylamide SDS-PAGE gels with (5 mm spacers) for 4 to 5 hours at 500 volts. The exact position of actins was verified by comigration with purified bovine actin (Sigma Co.) and prepackaged 2-D protein standards containing actin (Bio-Rad). Proteins were detected with silver stain applied per manufacturer recommendations (Genomic Solutions), dried, scanned, aligned, and quantitated (Melanie II, Version 2.2, Bio-Rad). Relative spot intensities were compared after matching for gel staining. For experiments in which immunoblotting was performed after 2-D gel electrophoresis, dry polyacrylamide strips (Immobiline DryStrip; ampholytes, pH 4.5–5.5, 18 cm, Amersham) were used to perform 2-D gel electrophoresis (per manufacturer recommendations), rather than tube gels.

### Immunoblot

Freshly isolated stellate cells or cultured cells were lysed, separated by SDS-PAGE, and transferred to nitrocellulose. Nonspecific binding was reduced by preincubation with TBS-T containing 5% bovine albumin (Sigma) and 2% serum (from the same species as the secondary antibody). Nitrocellulose blots were incubated overnight with *Acta2* antibody, or anti-cytoplasmic β actin antibody (Sigma), diluted 1∶2000 and washed 3 times with PBS. Bound primary antibody was detected following incubation with horseradish peroxidase conjugated anti-mouse IgG (Amersham), followed by ECL (Amersham Life Science). Bands were visualized on multiple exposures to autoradiography film (Eastman Kodak Co.) and data collected over a narrow range of X-ray film linearity and quantitated by scanning densitometry.

### Collagen lattice preparation and stellate cell contraction

Contraction assays were performed in 24-well flat-bottom tissue culture plates (Corning Glass Works) as previously described [65]. In brief, culture vessels were washed with PBS (Sigma) containing 1% bovine serum albumin (Sigma) and air-dried. A mixture of 8 parts Vitrogen (Celltrix Corp.), 1 part 10x MEM (Gibco BRL) and 1 part 0.2 M HEPES was added to each culture well, and allowed to gel. Cells isolated from normal or injured livers were layered on top of the collagen lattice and cultured for a specified time, after which mediators were added to induce contraction and lattices were detached by gentle circumferential dislodgment using a 200 µL micro-pipet tip. Contraction was monitored electronically as the change in lattice area over time.

### Antisense oligodeoxynucleotides, transfection

Hepatic stellate cells were isolated and cultured as above. Transfection of antisense or sense phosphorothioate deoxyoligonucleotides (oligos, Operon Technologies, Inc.,) was performed after cell attachment with lipofectin (Gibco BRL) or FuGENE/mL (Roche Diagnostics Co.) as per the manufacturers specifications. The oligo and transfection mix was replaced every 48 hours. Oligos were used at concentrations of 100 nM, 1 µM, or 10 µM. Antisense phosphorothioate oligos were directed at the translation start region (+16 to +30; 5′-CAG-AGC-TGT-GCT-GTC-3′), the mid portion of the gene in the coding region (+685 to +699, 5′-AGG-AGC-AGT-GGC-CAT-3′), and the 3′ untranslated region (+1204 to +1218; 5′-TCC-ACA-AAA-CAT-TCA-3′, termed 3′UT #1, and +1186 to 1205; 5′-CAC-AGT-TGT-GTG-CTA-GAG-AC-3′, termed 3′UT #2). Random (5′-ATG-TAG-TCA-CTT-CAA-3′) and specific sense (+1204 to +1218; 5′-TGA-ATG-TTT-TGT-GGA -3′) phosphorothioate oligonucleotides served as negative controls.

### siRNA knockdown

Hepatic stellate cells were as above. Cells were transduced with a specific siRNA to *Acta2* (siActa2): sense- ucAGAcAuGuGcuAcccuudTsdT, antisense- AAGGGuAGcAcAUGUCUGAdTsdT or a control siRNA to luciferase (siLuc): sense: 5′-cuuAcGcuGAGuAcuucGAdTsdT-3′ antisense: 5′- UCGAAGuACUcAGCGuAAGdTsdT (2′-O-methyl-modified nucleotides are in lower case; s, phosphorothioate linkage; dT, deoxythymidine) by using lipofectamie RNAimaxi (Invitrogen) for 48 hours according to the manufacturer's directions. Following 1 further day of culture in 0.5% serum medium, cells were harvested. Specific bands were quantitated and the raw volume of the control band(s) of *Acta2* or Erk1/2 (25 nM) were arbitrarily set at 100. Specific expression in each sample was presented as a relative percentage.

### Cell Proliferation

Cells were seeded in 96 well plates at 1×10^4^ cells per well and cultured for 2 days. On the third day of culture, cells were transduced with siActa2 or siLuc for 48 hours as above. Cell proliferation was measured by the MTS method (Promega) according to the manufacturer's instructions.

### Mouse embryo fibroblast isolation

Mouse embryo fibroblasts were isolated from mice with targeted deletion of *Acta2*, a kind gift from Dr. Robert Schwartz [30] as described [66]. In brief, embryos from heterozygote crosses were isolated at day 12–13 gestation, and each embryo was minced in 0.25% trypsin-EDTA (Gibco BRL). Cells were dispersed by shaking at 4°C for 2 hours, and then plated in DMEM containing 10% fetal bovine serum (Both from Gibco BRL). Cells were trypsinized and passed after 24 hours, and all experiments performed at passage 2–6.

### Statistics

ANOVA or Fisher's exact t tests were used for statistical comparisons. Each experiment utilized cells from a different animal. For calculation of mean values and statistical variation, “n” refers to the number of separate experiments each with an individual cell preparation. Error bars depict the standard error of the mean (SEM) unless stated otherwise; absence of error bars indicates that the SEM was less than 1%, unless stated otherwise.

## Supporting Information

Figure S1 **Actin isoforms - their amino acid variation and isoelectric points (pIs).** Each of the 6 actin isoforms is listed; GenBank accession numbers are provided, along with corresponding molecular sizes, amino acid numbers and pIs. The table also depicts a theoretical estimation of isoactin pIs by in silico analysis of the amino acid sequence, which was performed for each actin isoform as described [67] (see http://ca.expasy.org/tools/pi_tool.html).
(EPS)

Figure S2 ***Acta*** **antisense oligonucleotides inhibit cell motility (immunocytochemistry).** Stellate cells were as in Figure 5. Twenty-four hours after scratch wounding, cells were subjected to immunocytochemistry as in Figure 4. In (A, D, cytoplasmic β-actin) and (B, E, *Acta2*), representative images of cells exposed to 3′UT #1 sense oligonucleotides (A, B) and 3′UT #1 antisense oligonucleotides (D, E) are shown. In C (sense oligonucleotides) and F (antisense oligonucleotides), merged images are depicted in overlays. Representative areas from typical experiments are shown (n>12). Bar = 150 microns.
(EPS)
